# Safety, Tolerability, and Immunogenicity of Revaccination With mRNA-1345, an mRNA Vaccine Against Respiratory Syncytial Virus, Administered 12 Months Following a Primary Dose in Adults Aged ≥50 Years

**DOI:** 10.1093/cid/ciaf515

**Published:** 2025-09-24

**Authors:** Jaya Goswami, Jose F Cardona, Jorge Caso, Denise C Hsu, Alana K Simorellis, Lauren Wilson, Rakesh Dhar, Xiaowei Wang, Archana Kapoor, Avi Collins, Vinicius Righi, Lan Lan, Jiejun Du, Honghong Zhou, Sonia K Stoszek, Christine A Shaw, Caroline Reuter, Eleanor Wilson, Rituparna Das

**Affiliations:** Moderna, Inc., Cambridge, Massachusetts, USA; Indago Research and Health Center, Hialeah, Florida, USA; Suncoast Research Associates LLC, Miami, Florida, USA; Moderna, Inc., Cambridge, Massachusetts, USA; Moderna, Inc., Cambridge, Massachusetts, USA; Moderna, Inc., Cambridge, Massachusetts, USA; Moderna, Inc., Cambridge, Massachusetts, USA; Moderna, Inc., Cambridge, Massachusetts, USA; Moderna, Inc., Cambridge, Massachusetts, USA; Moderna, Inc., Cambridge, Massachusetts, USA; Moderna, Inc., Cambridge, Massachusetts, USA; Moderna, Inc., Cambridge, Massachusetts, USA; Moderna, Inc., Cambridge, Massachusetts, USA; Moderna, Inc., Cambridge, Massachusetts, USA; Moderna, Inc., Cambridge, Massachusetts, USA; Moderna, Inc., Cambridge, Massachusetts, USA; Moderna, Inc., Cambridge, Massachusetts, USA; Moderna, Inc., Cambridge, Massachusetts, USA; Moderna, Inc., Cambridge, Massachusetts, USA

**Keywords:** respiratory syncytial virus, safety, immunogenicity, mRNA RSV vaccine, revaccination

## Abstract

**Background:**

mRNA-1345 is a respiratory syncytial virus (RSV) vaccine approved for prevention of RSV-associated lower respiratory tract disease in individuals ≥ 60 years. Durability of efficacy is being evaluated in clinical trials. Data on revaccination in adults are needed.

**Methods:**

This open-label, phase 3 trial evaluated revaccination with 50 µg mRNA-1345 administered 12 months after primary vaccination in participants aged ≥50 years. The primary objectives were the immunogenicity (RSV-A and RSV-B neutralizing antibody [nAb] responses), tolerability, and safety of revaccination.

**Results:**

Overall, 543 participants were revaccinated. Most adverse reactions were mild/moderate (median duration, 2 days), with no new safety concerns identified. Coprimary immunogenicity endpoints met prespecified noninferiority criteria based on Day 29 geometric mean titer ratios (GMRs; revaccination vs primary vaccination). At Day 29, nAb GMRs (95% confidence interval [CI]) were 1.08 (1.0–1.17) for RSV-A and 0.91 (.84–.98) for RSV-B. Seroresponse rates (≥4-fold rise from baseline; 95% CI) at Day 29 were 77.5% (73.7–81.0) for RSV-A and 47.5% (43.2–51.9) for RSV-B, with a ≥2-fold rise in titers observed in 91.6% (88.9–93.8) and 69.8% (65.7–73.8) of participants, respectively. Following primary vaccination, RSV nAb titers increased by Day 29 and gradually declined over 12 months, yet remained above baseline levels. Revaccination at 12 months increased nAb titers, similar to the response observed after the primary dose.

**Conclusions:**

Revaccination of adults ≥ 50 years with mRNA-1345 was well tolerated, with a safety profile consistent with the primary dose. Respiratory syncytial virus nAb at Day 29 was noninferior to those after a primary mRNA-1345 dose, with antibody response persisting for 12 months.

**Clinical Trials Registration:**

ClinicalTrials.gov: NCT05330975.

## Teaser

This is a final analysis from a phase 3 study demonstrating the immunogenicity, tolerability, and safety of mRNA-1345, an mRNA vaccine targeting respiratory syncytial virus, when given as an open-label revaccination at 1 year following a primary dose in adults aged ≥50 years.

Respiratory syncytial virus (RSV) is an enveloped, single-stranded RNA virus of the *Pneumoviridae* family, comprising 2 subtypes, RSV-A and RSV-B. Respiratory syncytial virus is a significant cause of acute respiratory illness in older adults and is a major contributor to severe disease in adults with comorbidities, particularly chronic lung or heart diseases, placing them at higher risk compared to healthy adults [1–3]. Respiratory syncytial virus is estimated to have caused 5.2 million cases of infection, 470 000 hospitalizations, and 33 000 in-hospital deaths in 2019 in high-income countries among adults aged ≥60 years [4].

Vaccines for prevention of RSV-associated lower respiratory tract disease (LRTD) in older adults are currently licensed in many countries. The US Centers for Disease Control and Prevention recommends a single dose of RSV vaccine in adults aged ≥75 years and adults aged 60–74 years who are at increased risk of severe RSV disease [5]. Antibody responses, particularly neutralizing antibodies (nAbs), correlate with protection against RSV severe disease [6, 7]. While routine revaccination is not yet recommended [8], natural infection does not confer lifelong immunity [9]. Periodic revaccination may be needed to sustain antibody levels and protection, and clinical studies are evaluating optimal timing.

The mRNA-1345 vaccine (mRESVIA; Moderna, Inc.) is a lipid nanoparticle-encapsulated mRNA-based vaccine that encodes the RSV F-protein stabilized in the prefusion conformation, a conserved target for nAbs across both RSV subtypes [1, 2], and is approved for the prevention of RSV-LRTD in adults aged ≥60 years [10–12]. In a pivotal phase 3 trial, mRNA-1345 elicited robust nAb responses [13] and was efficacious against RSV-A and RSV-B with an acceptable safety and tolerability profile [14].

Safety and immunogenicity of mRNA-1345 revaccination were previously evaluated in a phase 1 trial in adults aged 65–79 years [15]. Here, we report primary Day 29 immunogenicity outcomes from a phase 3 trial, along with Day 181 and Day 361 immunogenicity and safety data through the end of study (EoS), assessing a 50-µg dose of mRNA-1345 administered 12 months after the initial vaccination in adults aged ≥50 years.

## METHODS

### Study Design and Participants

This phase 3, randomized, open-label, multicenter clinical trial was conducted in the United States (NCT05330975). Eligible participants received either mRNA-1345 or mRNA-1345 coadministered with mRNA-1273.214 (COVID-19 Spikevax; Moderna, Inc., Cambridge, Massachusetts) as their primary vaccination in an earlier portion of the study [16] and were assigned to receive revaccination with mRNA-1345 (50 μg) 12 months after primary vaccination. All study participants were followed for 12 months following revaccination. Eligible adults aged ≥50 years were enrolled, including those with comorbid medical conditions that increase the risk of severe RSV. A full list of eligibility criteria is provided in the Supplementary Methods.

The study was conducted according to the Good Clinical Practice guidelines of the International Council for Harmonisation of Technical Requirements for Pharmaceuticals for Human Use and ethical principles derived from the Declaration of Helsinki. The protocol was approved by all applicable institutional review boards. Written informed consent was obtained from each participant prior to enrollment.

### Objectives

The primary safety objective was to evaluate the safety and tolerability of revaccination with mRNA-1345. The primary immunogenicity objective was to demonstrate noninferiority of immune response against RSV-A and RSV-B based on geometric mean titer ratios (GMRs) of nAbs on Day 29 following revaccination compared with nAbs on Day 29 following the primary dose. Secondary immunogenicity objectives included the assessment of geometric mean titers (GMTs) and geometric mean fold rises (GMFRs) of RSV-A and RSV-B nAbs from prerevaccination baseline through Day 361/EoS. Proportion of participants with seroresponse (defined as ≥4-fold increase in nAb titers from baseline prior to primary dose) against RSV-A and RSV-B and proportion of participants with ≥2-fold increase from baseline prior to primary dose up to revaccination Day 361/EoS were also included as secondary immunogenicity objectives. Additional information is provided in the Supplementary Methods.

### Study Interventions and Procedures

mRNA-1345 was administered as an intramuscular injection into the deltoid muscle.

Serum samples for immunogenicity assessments were collected on the day of revaccination (revaccination Day 1 preinjection), as well as Day 29 and Day 181 after revaccination, and at EoS (Day 361). Serum nAbs to RSV-A and RSV-B were measured using validated microneutralization assays [13, 16]. Solicited local and systemic adverse reactions (ARs) with onset within 7 days after each vaccination were recorded using electronic diaries. Unsolicited adverse events (AEs) were reported through 28 days. Medically attended AEs (MAAEs) were assessed from revaccination Day 1 through Day 181. Serious AEs (SAEs), AEs of special interest (AESIs), and AEs leading to discontinuation from study participation were assessed from revaccination Day 1 through EoS (up to Day 361). Further details on immunogenicity and safety assessments are provided in the Supplementary Methods.

### Statistical Analysis

The planned sample size of 500 participants was selected to ensure 99% and 92% probability of observing at least 1 AE if the true event rate was 1% and 0.5%, respectively, and to provide approximately 86.6% and 98.3% power to demonstrate noninferiority of mRNA-1345 revaccination to RSV-A and RSV-B, respectively. Safety analyses were based on the safety set (all participants who received mRNA-1345 revaccination), and summaries of solicited ARs were based on the solicited safety set (all participants who received mRNA-1345 revaccination and contributed any solicited AR data).

The primary immunogenicity analyses were performed using the per-protocol set, which included all participants who were revaccinated with mRNA-1345, had a prerevaccination immunogenicity assessment and at least 1 after-revaccination immunogenicity assessment at revaccination Day 29, complied with the immunogenicity testing schedule, and had no major protocol derivations that impact key or critical data. The coprimary endpoints for RSV-A and RSV-B were based on the comparison of GMT at revaccination Day 29 and primary vaccination Day 29. Noninferiority of the GMT at revaccination Day 29 relative to Day 29 following primary dose was demonstrated if the lower bound (LB) of the 95% confidence interval (CI) for the GMR exceeded 0.667 (ie, LB > 0.667), corresponding to a noninferiority margin of 1.5. Geometric mean titer ratio was the ratio of the GMT of nAbs at revaccination Day 29 over GMT of nAbs at Day 29 following primary dose. The GMR and the 95% CI were calculated by back-transforming the mean and 95% CI of paired difference of nAbs on the logarithmic scale (based on t-distribution) between revaccination Day 29 and Day 29 following primary dose within each participant. Secondary endpoints on seroresponse rates (SRRs) for RSV-A and RSV-B nAbs at revaccination Day 29 were provided with 2-sided 95% CIs using the Clopper–Pearson method. The SRR difference and 95% CI between Day 29 following revaccination and Day 29 after primary dose was also calculated alongside 2-sided 95% CIs using a linear probability model with repeated measures (via PROC GENMOD in SAS). Immunogenicity was also analyzed by prespecified participant subgroups, including age, sex, race, and ethnicity. All statistical analyses were performed using SAS version 9.4 software.

## RESULTS

### Participants

**Figure 1.**
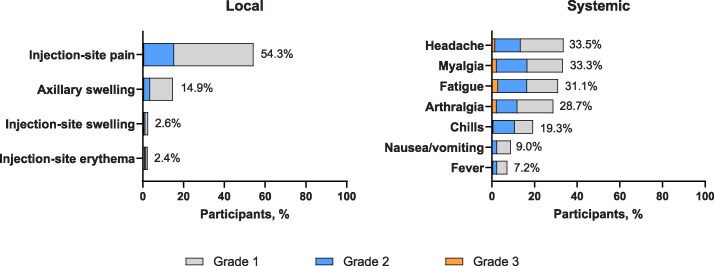
Solicited adverse reactions within 7 d after mRNA-1345 revaccination (solicited safety set). There were no local or systemic Grade 4 ARs. Abbreviations: AR, adverse reaction.

The study enrolled participants for revaccination between 25 August and 24 October 2023 across 37 sites in the United States, with participants at 36 sites receiving revaccination. Overall, 543 participants were revaccinated with mRNA-1345 and were included in the safety set. The 18 study discontinuations across vaccination groups were mainly due to loss to follow-up (Supplementary Table 1 and Figure 1). The per-protocol set included 525 participants.

At baseline (prior to primary vaccination), the median (range) age of the participants was 62 (50–91) years; participants aged ≥60 years were well represented (61.3%). Overall, 58% of participants were female, 76% were White, 20% were Black, and 47% were Hispanic or Latino (Table 1). The median (range) interval between primary vaccination and revaccination was 384 days (range: 365–444 days) or 12.6 months.

**Table 1.** Baseline Demographics of Study Participants (Safety Set)

Characteristic	Overall mRNA-1345 Revaccination Group (N = 543)
Age at enrollment, y	
Mean (standard deviation)	62.3 (7.39)
Median (min, max)	62.0 (50, 91)
Age group, n (%)^a^	
50–59 y	210 (38.7)
60–74 y	295 (54.3)
≥60 y	333 (61.3)
≥75 y	38 (7.0)
Sex, n (%)	
Male	230 (42.4)
Female	313 (57.6)
Race, n (%)	
White	412 (75.9)
Black	107 (19.7)
Asian	7 (1.3)
Other^b^	17 (3.1)
Ethnicity, *n* (%)	
Hispanic or Latino	254 (46.8)

Percentages are based on the number of participants in the safety set.

^a^Age groups are derived based on the age provided by the investigators on the case report form.

^b^Other race includes American Indian or Alaska Native, Native Hawaiian or other Pacific Islander, other, multiple, or not reported.

### Safety

The median (range) duration of follow-up from revaccination was 354 days (28–414). Any solicited local and systemic ARs within 7 days after revaccination were reported by a respective 55.8% and 50.3% of participants, were primarily Grade 1 or 2 in severity, and had a median time to onset and duration of 2 days (Supplementary Table 2). Injection-site pain (54.3%) was the most frequently reported solicited local AR (Figure 1). Headache (33.5%), myalgia (33.3%), fatigue (31.1%), and arthralgia (28.7%) were the most frequently reported (≥20%) solicited systemic ARs. Grade 3 solicited ARs were infrequent; of those reported, the most frequent Grade 3 solicited local AR was injection-site erythema (1.1%), and fatigue was the most frequent Grade 3 solicited systemic AR (2.9%). There were no Grade 4 solicited local or systemic ARs. Solicited ARs were reported at similar overall frequency and severity across age groups (Supplementary Table 3).

Up to 28 days following revaccination, unsolicited AEs were reported in 5.7% of revaccinated participants (Supplementary Table 4). Serious AEs were infrequent (0.6% of participants) and none were considered by the investigator to be related to the vaccine. There were no fatal events or AEs leading to discontinuation reported within 28 days after revaccination. An AESI of thrombocytopenia was reported on Day 28 in 1 participant who had multiple comorbidities; the event was considered unrelated to mRNA-1345 per the investigator. Nonserious related events were reported by 5 participants and included reactogenicity type events.

Up to the Day 361/EoS, there were 28 SAEs (5.2%), 3 fatal events, and 5 AEs leading to discontinuation; none of the serious AEs, fatal events, AESIs, or AEs leading to discontinuation were assessed as related by the investigator (Supplementary Table 4). There was 1 additional AESI of thrombocytopenia reported on Day 168 in 1 participant with concurrent cellulitis; the event was considered unrelated to mRNA-1345 per the investigator. One case of acute pericarditis, adjudicated by an independent cardiac monitoring committee and reported as acute pericardial effusion with onset on Day 246, was assessed by the investigator as not related to mRNA-1345. There were no events of anaphylaxis, Guillain–Barré syndrome, acute disseminated encephalomyelitis, or Bell's palsy/facial paralysis reported in revaccinated participants.

### Immunogenicity

**Figure 2.**
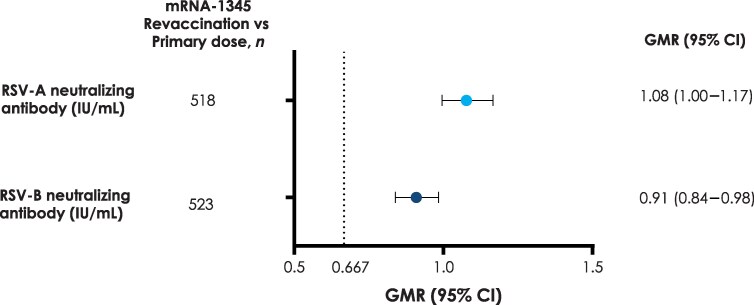
Neutralizing antibody GMRs of mRNA-1345 revaccination versus primary dose for RSV-A and RSV-B in adults aged ≥50 y at Day 29 following revaccination (per-protocol set). Noninferiority for GMT was demonstrated if the lower bound of the 95% CI of the GMR was >0.667 (dotted line; revaccination vs primary dose). The GMR and the 95% CI were calculated by back-transforming the mean and 95% CI of the paired difference of nAbs on the logarithmic scale (based on t-distribution) between revaccination Day 29 and Day 29 following the primary dose within each participant. N is the number of participants with nonmissing antibody data at baseline (primary dose Day 1), primary dose Day 29, and revaccination Day 29. Abbreviations: CI, confidence interval; GMR, geometric mean titer ratio; GMT, geometric mean titer; IU, international units; nAb, neutralizing antibody; RSV, respiratory syncytial virus.

Noninferiority of the immune response to RSV-A and RSV-B antigens after revaccination compared to after primary vaccination was demonstrated for both coprimary endpoints, as the LB of the 2-sided 95% CI of the GMR exceeded 0.667. At Day 29, the GMR for RSV-A nAb was 1.08 (95% CI: 1.00–1.17) and for RSV-B nAb was 0.91 (95% CI: .84–.98) (Figure 2; Supplementary Table 5).

**Figure 3.**
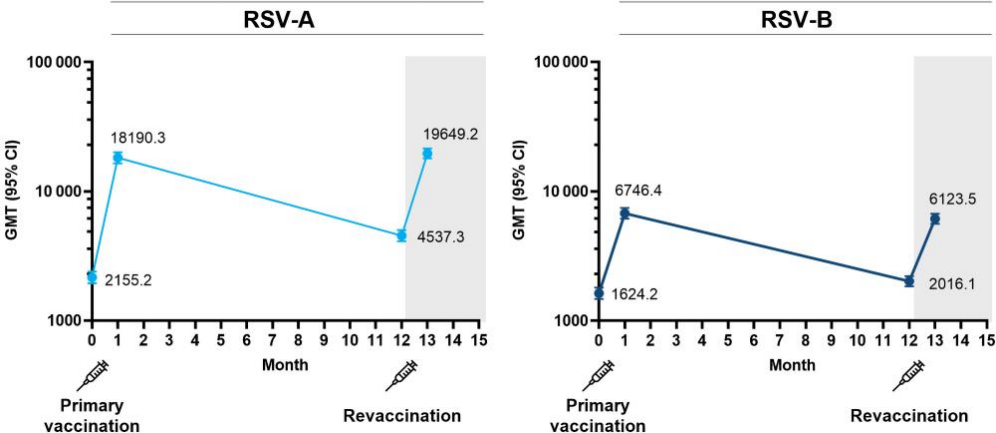
Neutralizing antibody GMTs following mRNA-1345 primary dose and revaccination at 12 m (per-protocol set). GMTs (IU/mL) with corresponding 95% CIs are presented for RSV-A and RSV-B before primary vaccination (baseline) and 28 d after the primary mRNA-1345 50-μg dose (Day 29 or Month 1); gray shading denotes GMTs at 12 m after the primary dose (prior to revaccination) and at 1 m after mRNA-1345 50-μg revaccination (12 m after primary dose). Abbreviations: CI, confidence interval; GMT, geometric mean titer; IU, international units; nAb, neutralizing antibody; RSV, respiratory syncytial virus.

Neutralizing antibody GMTs (IU/mL) for both RSV-A and RSV-B increased at Day 29 after primary vaccination with mRNA-1345 (Figure 3; Supplementary Table 6). Twelve months following primary vaccination, the GMTs for RSV-A remained above baseline, reflecting an immunogenicity response with a GMFR of 2.10 (95% CI: 1.92–2.30); the GMFR for RSV-B at 12 months following primary vaccination was 1.24 (95% CI: 1.15–1.34). Revaccination with mRNA-1345 at 12 months elicited RSV-specific antibody responses similar to those after the primary dose (Figure 3; Supplementary Table 6). At Day 29 following primary vaccination, the RSV-A nAb GMFR from baseline was 8.46 (95% CI: 7.63–9.38), and RSV-B nAb GMFR from baseline was 4.16 (95% CI: 3.78–4.57; Supplementary Table 6). At Day 29 after revaccination, both RSV-A and RSV-B GMTs increased and the GMFRs were similar to those observed after primary vaccination. The RSV-A GMFR from baseline was 9.11 (95% CI: 8.26–10.04). For RSV-B, the GMFR from baseline to Day 29 after revaccination was 3.77 (95% CI: 3.43–4.14; Supplementary Table 6). Neutralizing antibody levels persisted above baseline through 12 months following revaccination. The RSV-A GMFR at 12 months after revaccination from baseline was 2.26 (95% CI: 2.06–2.48). For RSV-B, the GMFR from baseline was 1.42 (95% CI: 1.31–1.53).

Although noninferiority of SRR was not specified as an endpoint, the SRR difference for RSV-A was 2.1% (95% CI: −1.7 to 5.9) and for RSV-B was −0.6% (95% CI: −5.0 to 3.9), both exceeding a LB of −10%. Seroresponse rates for RSV-A and RSV-B (secondary endpoints) at Day 29 after revaccination were 77.5% (95% CI: 73.7–81.0) and 47.5% (95% CI: 43.2–51.9) of participants, respectively (Supplementary Table 6). A ≥2-fold increase from baseline in nAb titers at Day 29 after revaccination was seen in 91.6% (95% CI: 88.9–93.8) of participants for RSV-A and for 69.8% (95% CI: 65.7–73.8) of participants for RSV-B. In addition, revaccination with mRNA-1345 increased antibody responses among participants with a wide range of prerevaccination antibody titers (Supplementary Figure 2). Participants were grouped into quartiles based on their nAb titers before revaccination; those in the lowest quartile had the lowest prerevaccination nAb while those in the highest had the highest prerevaccination nAb. All quartile groups demonstrated an increase in titers, with the participants in the lowest quartile of prerevaccination titers having the greatest difference in titers following revaccination compared to participants in the higher quartiles.

**Figure 4.**
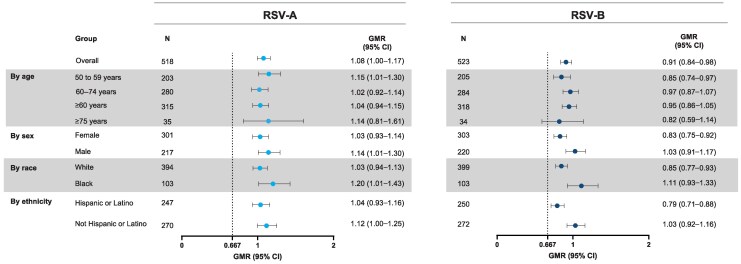
Subgroup analysis of RSV-A and RSV-B nAb responses at Day 29 after mRNA-1345 revaccination (per-protocol set). Age group was based on the age at the time of informed consent in the case report form at the start of the trial. The GMR and its 95% CI were calculated by back-transforming the mean and 95% CI of the paired difference of nAbs on the logarithmic scale (based on t-distribution) between revaccination Day 29 and Day 29 following the primary dose within each participant. Noninferiority was demonstrated if the lower bound of the 95% CI of the GMR exceeded 0.667 (dotted line). The subgroup analysis was not powered for meeting noninferiority criteria. Abbreviations: CI, confidence interval; GMR, geometric mean titer ratio; nAb, neutralizing antibody; RSV, respiratory syncytial virus.

RSV-A and RSV-B nAb responses following revaccination were evaluated across prespecified subgroups (including age, sex, race, and ethnicity). RSV-A and RSV-B GMRs (revaccination Day 29 vs primary dose Day 29) were generally consistent in the prespecified subgroups (Figure 4). Of interest, in adults aged ≥60 years, GMR was 1.04 (95% CI: .94–1.15) for RSV-A and 0.95 (95% CI: .86–1.05) for RSV-B, consistent with the responses in the overall cohort. In participants ≥ 75 years, GMRs were 1.14 (95% CI: .81–1.61) and 0.82 (95% CI: .59–1.14) for RSV-A and RSV-B, respectively, though the sample size was limited (Figure 4).

## DISCUSSION

This open-label, phase 3 study evaluated the safety and immunogenicity of revaccination with 50 μg of mRNA-1345, the licensed dose of RSV vaccine, in adults ≥ 50 years of age, administered 12 months after the primary vaccination. The study met the prespecified noninferiority success criteria for both primary immunogenicity endpoints. Revaccination was well tolerated, with no new safety concerns identified, and the frequency and severity of solicited ARs were similar to those observed following primary vaccination (Supplementary Tables 2 and 3).

RSV-A and RSV-B nAb titers remained elevated above baseline through 12 months after primary vaccination. Revaccination at 12 months elicited nAb GMTs at Day 29 that were noninferior to those observed after the primary mRNA-1345 dose. Neutralizing antibody levels also persisted through 12 months following revaccination. Across all baseline titers, mRNA-1345 effectively increased responses against both RSV subtypes. Notably, even participants with the highest prevaccination titers demonstrated increased titers following revaccination, highlighting the ability of mRNA-1345 to induce responses regardless of baseline immunity. Based on these findings, revaccination with mRNA-1345 is expected to confer vaccine efficacy comparable to that of the primary dose.

RSV-A and RSV-B nAb responses following revaccination were consistent across age, sex, race, and ethnicity subgroups and were similar to those observed in the overall cohort, suggesting that revaccination with mRNA-1345 confers similar benefit across subgroups. In particular, periodic revaccination with mRNA-1345 may be beneficial for older adults, who are at increased risk of severe RSV disease and its associated morbidity and mortality.

Respiratory syncytial virus is a major respiratory pathogen that disproportionately affects infants, adults with cardiorespiratory disease, older adults, and immunocompromised individuals, contributing to significant morbidity and mortality worldwide [17, 18]. Lifelong immunity is not conferred by RSV infection [19], and vaccine efficacy against RSV-LRTD decreases over time [20–22]. Declining vaccine efficacy and seasonal RSV epidemics underscore the need to evaluate revaccination, particularly in vulnerable populations at high risk of severe outcomes.

Clinical trial data from both adjuvanted and nonadjuvanted protein subunit RSV vaccines indicate that immune responses following revaccination at intervals of 1, 2, and 3 years are not restored to levels observed after primary vaccination [23–25]. A second dose of an adjuvanted preF protein subunit RSV vaccine given 12 months after primary vaccination conferred no added benefit through 2 RSV seasons [8]. In contrast, data presented here of an mRNA-based RSV vaccine suggest that revaccination elicits immune responses comparable to those observed after the primary series. Neutralizing antibodies have been shown to correlate with efficacy conferred by mRNA-1345 [7]. These data suggest that revaccination with mRNA-1345 at 12 months will confer similar efficacy as primary vaccination.

Several factors likely contribute to observed variations in antibody responses to revaccination across different RSV vaccine platforms. Differences in immune responses reported across various studies may partially result from assay variability. This heterogeneity limits the ability to make direct cross-study comparisons. Notably, neutralization assay outcomes in this study and related mRNA-1345 studies are reported using the WHO international standard for RSV antisera (IU/mL). To more accurately evaluate the relative immune responses after revaccination induced by distinct vaccine platforms, further research employing standardized and harmonized assay approaches is needed [26].

Structural variations in antigen design among the three approved RSV vaccines may influence immune recall and revaccination response. For instance, vaccine-induced immune interference (previously described in HIV and adenoviral-based vaccines) can occur when additional immune responses are directed against vaccine components other than the intended antigen [27, 28]. Protein subunit RSV vaccines include a foldon protein construct (T4-phage fibritin trimerization domain) to stabilize the preF trimeric antigen, an approach essential for inducing nAb responses against RSV [29]. Preclinical and early clinical studies demonstrate that RSV preF vaccines containing the foldon construct can induce anti-foldon antibodies, which may attenuate increasing responses upon subsequent revaccination [30, 31]. However, recent data show that anti-foldon antibodies induced in humans after administration of an adjuvanted preF protein subunit vaccine do not appear to interfere with RSV-neutralizing responses [31]. Thus, the clinical implications of anti-foldon antibodies remain unclear. Conversely, the preF protein encoded by mRNA-1345 does not contain a foldon domain and therefore would not induce an anti-foldon antibody response.

No safety concerns were identified following revaccination with mRNA-1345. Revaccination at 12 months after the primary dose was well-tolerated, with reactogenicity comparable to that observed in the pivotal trial [14]. Solicited ARs were mostly mild to moderate and transient.

Study limitations included the relatively small number of participants aged ≥75 years. A phase 3 extension study is underway to assess the immunogenicity and safety of mRNA-1345 revaccination at 24 months after primary vaccination (NCT05127434).

In conclusion, this open-label, phase 3 trial demonstrated that revaccination with 50 μg of mRNA-1345 in adults aged ≥50 years was immunogenic and showed no safety concerns. These results demonstrate that revaccination with mRNA-1345 as early as 1 year after primary vaccination elicits immune responses that are comparable to those elicited following primary vaccination and is therefore expected to provide comparable vaccine efficacy to that following primary vaccination.

## Supplementary Material

ciaf515_Supplementary_Data

## Supplementary Data

Supplementary materials are available at *Clinical Infectious Diseases* online. Consisting of data provided by the authors to benefit the reader, the posted materials are not copyedited and are the sole responsibility of the authors, so questions or comments should be addressed to the corresponding author.
