# Supplementary material for: Safety, Tolerability, and Immunogenicity of Revaccination With mRNA-1345, an mRNA Vaccine Against Respiratory Syncytial Virus, Administered 12 Months Following a Primary Dose in Adults Aged ≥50 Years
Source: Clin Infect Dis. 2025 Sep 24;82(4):e855–62. doi: 10.1093/cid/ciaf515 (PMC13131948; doi:10.1093/cid/ciaf515)
Supplement: ciaf515_Supplementary_Data [file ciaf515_supplementary_data.docx]

**Supplementary Material**

**Safety, Tolerability, and Immunogenicity of Revaccination with mRNA-1345, an mRNA Vaccine Against RSV, Administered 12 Months Following a Primary Dose in Adults Aged ≥50 Years**

This appendix has been provided by the authors to give readers additional information.

**List of trial investigators**

| **PI name** | **Institution** | **Location** |
| --- | --- | --- |
| Akinsola, Adebayo | Tekton Research – Georgia – Platinum – PPDS | Chamblee, GA |
| Downey, H. Jackson | Westside Center for Clinical Research | Jacksonville, FL |
| Bell, Robert Codey | Tekton Research - Beaumont - Platinum - PPDS | Beaumont, TX |
| Anderson, Rachel Lacy | Primary Health Partners | Moore, OK |
| Vutikullird, Apinya Bee | Ark Clinical Research – Tustin –  Clinedge – PPDS | Long Beach, CA |
| Parikh, Naresh | Georgia Clinic / CCT Research | Norcross,GA |
| Patel, Suchet | Meridian | Endwell, NY |
| Cardona, Jose F. | New Phase Research & Development | Hialeah, FL |
| Caso, Jorge | Suncoast Research Associates LLC – ERN – PPDS | Miami, FL |
| Chu, Laurence | Benchmark Research – Austin – HyperCore - PPDS | Austin, TX |
| De La Cruz, Luis | Velocity Clinical Research – Greenville – ERN – PPDS | Greenville, SC |
| Fussell, Suzanne | Long Beach Clinical Trials, LLC | Long Beach, CA |
| Kutner, Mark | Suncoast Research Group LLC – ERN-PPDS | Miami, FL |
| Logan, Douglas | Meridian Clinical Research – Cincinnati – Platinum – PPDS | Springdale, OH |
| Pearce, Katherine | Meridian Clinical Research (Baton Rouge-Louisiana) – Platinum – PPDS | Baton Rouge, LA |
| Essink, Brandon | Meridian Clinical Research | Omaha, NE |
| Soufer, Joseph | Chase Medical Research LLC | Waterbury, CT |
| Brosz, Adam | Meridian Clinical Research (Grand Island) – Platinum – PPDS | Grand Island, NE |
| Ensz, David | Meridian Clinical Research (Sioux City – Iowa) | Sioux City, IA |
| Beckes, John | Acclaim Clinical Research | San Diego, CA |
| Espinosa-Fernandez, Ivette | Revival Research Corporation – Clinedge – PPDS | Doral, FL |
| Kalen, Vicki MD | Del Sol Research Management – Clinedge - PPDS | Tucson, AZ |
| Navarro, Martha | Ark Clinical Research – Long Beach – Clinedge – PPDS | Long Beach, CA |
| Sanchez, William MD | Floridian Clinical Research – ClinEdge – PPDS | Miami Lakes, FL |
| Siddiqui, Farhan | Velocity Clinical Research – Spartanburg – ERN – PPDS | Spartanburg, SC |
| Eagerton, Donald | Trial Management Assoc. | Myrtle Beach, SC |
| Lorch, Daniel | Teradan Clinical Trials | Brandon, FL |
| Clarke, Natalie | New Phase Research & Development | Knoxville, TN |
| Marquez-Mendoza, Otto | Dolphin Medical Research | Doral, FL |
| Amin, Faisal | Central Valley Research, LLC | Modesto, CA |
| Patel, Rahul | Meridian Clinical Research (Rockville Maryland) – Platinum – PPDS | Rockville, MD |
| Heller | Long Beach Clinical Trials, LLC | Long Beach, CA |
| Myneni, Banu | Meridian Clinical Research – Family Practice Ports – Portsmouth – Platinum – PPDS | Portsmouth, VA |
| Simmons, Todd | Medical Center For Clinical Research - M3 WR - ERN - PPDS | San Diego, CA |
| Paliwal, Amit | Empire Clinical Research | Pomona, CA |
| Ward, Derrick | Meridian, Overland Park | Overland Park, KS |
| Blad, Kenneth | Midwest Regional Health Services, LLC | Omaha, NE |
| Peterson, Bryce | Cope Family Medicine | Boutiful, UT |
| Pirani, Aziz | Lifeline Primary Care, Inc | Lilburn, GA |
| Peterson, Michael | Springville Dermatology | Springville, UT |
| Harper, Charles | Velocity Clinical Research (Norfolk – Nebraska) – PPDS | Norfolk, NE |
| Pervaiz, Syed | Santa Rosa Medical Centers of Nevada | Las Vegas, NV |

**Supplementary Methods**

**Study Design**

This study was designed as a single arm, open-label study to evaluate safety, tolerability, and immunogenicity of revaccination with mRNA-1345 in adults aged ≥50 years who had previously received a primary dose of mRNA-1345. Eligible participants received revaccination with mRNA-1345 at 1 year following the primary dose. A total of 543 participants from the primary vaccination of the study received revaccination; participants came from 2 cohorts in the primary vaccination: those who received mRNA-1345 plus COVID-19 vaccine (mRNA-1273.214) as primary vaccination on Day 1 and those who received mRNA-1345 plus placebo as primary vaccination on Day 1.

**Study Inclusion Criteria**

Participants were eligible to be included in the study only if all the following criteria applied:

1. Participants at study sites who had been enrolled in the previous part of the study (primary vaccination); had immunogenicity blood sampling at primary vaccination baseline and Day 29; completed the Day 211/EoS visits for primary vaccination; were included in the per protocol (PP) set; and received 1 dose of mRNA-1345 at least 12 months (but no later than 15 months) prior to the time of enrollment
2. The investigator assessed that the participant understood and was willing and physically able to comply with protocol-mandated follow-up, including all procedures, and was in general good health according to the investigator's assessment
3. Female participants of nonchildbearing potential could be enrolled in the study. Nonchildbearing potential was defined as bilateral tubal ligation >1 year prior to screening, bilateral oophorectomy, hysterectomy, or menopause. A follicle-stimulating hormone level could have been measured at the discretion of the investigator to confirm menopausal status
4. Female participants of childbearing potential could be enrolled in the study if the participant: (1) had a negative urine pregnancy test at screening and on the day of injection, (2) had practiced adequate contraception or had abstained from all activities that could lead to pregnancy for 28 days prior to vaccination, (3) had agreed to continue adequate contraception through 3 months following revaccination and (4) was not currently breastfeeding. Adequate contraception was defined as the consistent and correct use of an approved contraceptive method in accordance with the product label
5. Participants were willing and able (on both a physical and cognitive basis) to provide informed consent prior to study enrollment
6. Participants were able to comply with study requirements, including having access to transportation for study visits
7. Participants had access to inbound and outbound telephone communication with caregivers and study staff

**Study Exclusion Criteria**

Participants were not eligible to be included in the study if any of the following criteria applied:

1. Participation in another interventional clinical research study where the participant had received any investigational product (drug/biologic/device) within 6 months before the planned date of the revaccination Day 1 study injection. Any prior receipt of an investigational or approved vaccine against RSV, except as part of mRNA-1345 Study P302 primary vaccination, was exclusionary
2. The participant was acutely ill or febrile (temperature ≥38.0℃ [100.4°F]) within 72 hours prior to the Screening Visit or revaccination Day 1. Participants meeting this criterion could have been rescheduled within the 14-day screening window and would have retained their initially assigned participant number
3. A history of a diagnosis or condition that, in the judgment of the investigator, was clinically unstable or could have affected participant safety, assessment of safety endpoints, assessment of immune response, or adherence to study procedures. Clinically unstable was defined as a diagnosis or condition requiring significant changes in management or medication within 60 days prior to screening and included ongoing workup of an undiagnosed illness that could lead to a new diagnosis or condition
   1. Asymptomatic conditions and conditions with no evidence of end-organ involvement (eg, mild hypertension, dyslipidemia) were not exclusionary if they were being appropriately managed and were clinically stable (ie, unlikely to result in symptomatic illness within the time course of the study)
   2. Illnesses or conditions could have been exclusionary, even if otherwise stable, because of therapies used to treat them (eg, immune-modifying treatments), at the discretion of the investigator
   3. Participants who had undergone surgical procedures within 7 days prior to revaccination Day 1 or were scheduled to undergo a surgical procedure within 28 days after the study injection were also excluded. However, minor surgical procedures under local anesthesia (eg, excision of a skin lesion) or diagnostic procedures (eg, colonoscopy) were allowed
4. A reported history of congenital or acquired immunodeficiency, asplenia or recurrent infections, or an immunocompromising/immunosuppressive condition that required systemic treatment with immunosuppressive therapy, including cytotoxic agents, radiotherapy, or monoclonal antibodies for cancer or an autoimmune disorder, with the following exceptions:
   1. Participants who were HIV-positive with a CD4 count ≥350 cells/mm³ and an undetectable HIV viral load within the past year (low-level variations up to 500 viral copies, which did not lead to changes in antiretroviral therapy) as determined from the participant’s medical records were permitted
   2. Certain immune-mediated conditions that were stable and well-controlled (eg, alopecia areata, Hashimoto thyroiditis, type 1 diabetes mellitus, gout, autoimmune ovarian failure) and those not requiring systemic immunosuppressants (eg, asthma, psoriasis, or vitiligo) were permitted at the discretion of the investigator
5. Participants who had received systemic immunosuppressants for more than 14 days in total within 180 days prior to the Revaccination Screening Visit (for glucocorticoids, ≥10 mg/day of prednisone or equivalent) or were anticipating the need for systemic immunosuppressive treatment at any time during the study (including intra-articular steroid injections) were excluded. Inhaled, nasal, and topical steroids were allowed
6. Participants with dermatologic conditions that could have affected local solicited adverse reaction (AR) assessments (eg, tattoos, psoriasis patches affecting the skin over the deltoid areas) were excluded
7. Participants with any reported history of anaphylaxis or known immediate hypersensitivity to any component of the study product or any mRNA vaccine(s), including polyethylene glycol, were excluded
8. Participants with a reported history of coagulopathy or a bleeding disorder that was considered a contraindication to intramuscular injection or phlebotomy were excluded
9. Participants with any medical, psychiatric, or occupational condition, including a reported history of drug or alcohol abuse, that, in the investigator’s opinion, might have posed additional risk due to participation in the study or interfered with the interpretation of study results were excluded
10. Participants with a known uncontrolled disorder of coagulation were excluded
    1. Note: Participants with well-controlled atrial fibrillation on prophylaxis for cardiovascular thromboembolism or stroke with medications such as aspirin, clopidogrel, prasugrel, dipyridamole, dabigatran, apixaban, rivaroxaban, or warfarin were allowed
11. Participants who had received or planned to receive any vaccine authorized or approved by a local health agency within 28 days prior to the study injection (revaccination Day 1) or planned to receive such a vaccine within 28 days after the study injections were excluded
12. Participants with a history of a serious reaction to any prior vaccination or Guillain-Barré syndrome occurring within 6 weeks after any prior influenza immunization were excluded
13. Participants who had received systemic immunoglobulins or blood products within 90 days prior to the Screening Visit or planned to receive systemic immunoglobulins or blood products during the study were excluded
14. Participants with a history of myocarditis, pericarditis, or myopericarditis within 2 months prior to screening were excluded. Participants who had not returned to baseline after their convalescent period were also excluded
15. Participants who had donated ≥450 mL of blood products within 28 days prior to the Screening Visit or planned to donate blood products during the study were excluded
16. Participants who were immediate family members or household members of study personnel, study-site staff, or sponsor personnel were excluded

**Immunogenicity Assessments**

Blood sample collection for humoral immunogenicity occurred on revaccination Day 1, revaccination Day 29, revaccination Day 181, and revaccination Day 361/EoS. The samples were processed and analyzed per the Laboratory Manual. Immunogenicity assessments were performed for all participants. RSV-A and RSV-B nAbs were measured by validated microneutralization assay [13, 16] and performed in a laboratory designated by the Sponsor.

**Safety Assessments**

Participants reported solicited local and systemic ARs in a provided electronic diary (eDiary) from revaccination Day 1 through revaccination Day 7 (the day of injection and the following 6 days). Solicited local ARs included injection site pain, injection site erythema (redness), injection site swelling/induration (hardness), and axillary (underarm) swelling or tenderness ipsilateral to the side of injection. Solicited systemic ARs included headache, fatigue, myalgia, arthralgia, nausea/vomiting, chills, and fever. Severity of solicited ARs was based on the toxicity grading scale for healthy adult and adolescent volunteers enrolled in preventive vaccine clinical trials.

Study investigators reported all unsolicited adverse events (AEs) and assessed the casualty (ie, if there was a reasonable possibility that vaccination caused the event). Severity of unsolicited AEs was determined by the investigator based on medical judgment and definitions of severity: mild (events not interfering with the participant’s daily activities), moderate (events causing some interference with the participant’s daily activities and requiring limited or no medical intervention), or severe (events preventing the participant’s daily activity and requiring intensive therapeutic intervention). AEs of special interest (AEs) included thrombocytopenia, anaphylaxis, myocarditis/pericarditis, and new onset of or worsening of neurologic disease (Neurologic diseases include the following: Guillain-Barré syndrome, acute disseminated encephalomyelitis, Bell’s palsy, and seizures including but not limited to febrile seizures and/or generalized seizures/convulsions). A complete physical examination, including vital sign measurement, was conducted at screening and on revaccination Day 1.

**Statistical Analysis**

The immunogenicity primary objectives were to evaluate the effect of revaccination with mRNA-1345 on the immune response to RSV-A and RSV-B.

**Co-primary endpoints based on GMT at revaccination Day 29**

The null hypothesis H0: immunogenicity response to revaccination of mRNA-1345, as measured by GMT at revaccination Day 29 using RSV-A or RSV-B nAb assay, was inferior to the GMT at Day 29 following primary dose. The noninferiority of the geometric mean titer (GMT) at revaccination Day 29 was demonstrated by the lower bound (LB) of the 95% CI for the geometric mean ratio (GMR), which ruled out 0.667 (i.e., LB>0.667) using a noninferiority margin of 1.5. GMR was the ratio of GMT of RSV-A or RSV-B nAbs at revaccination Day 29 over GMT of RSV-A or RSV-B nAbs at Day 29 following primary dose, calculated as the back transformation of mean of paired difference of RSV-A or RSV-B nAbs on the logarithmic scale (based on t-distribution) between revaccination Day 29 and Day 29 after the primary dose.

**Supplementary Figures**

**Figure S1.** Participant Disposition (Enrolled Set).


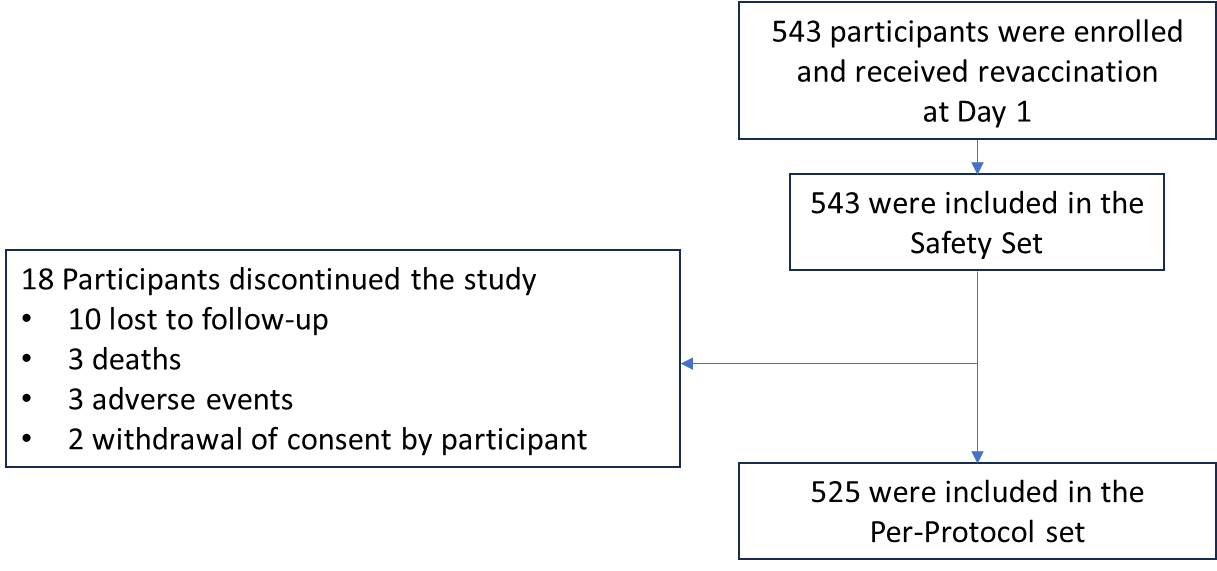


**Figure S2.** Neutralizing Antibody Responses Following mRNA-1345 Revaccination by Pre-Revaccination GMT (Quartiles)​


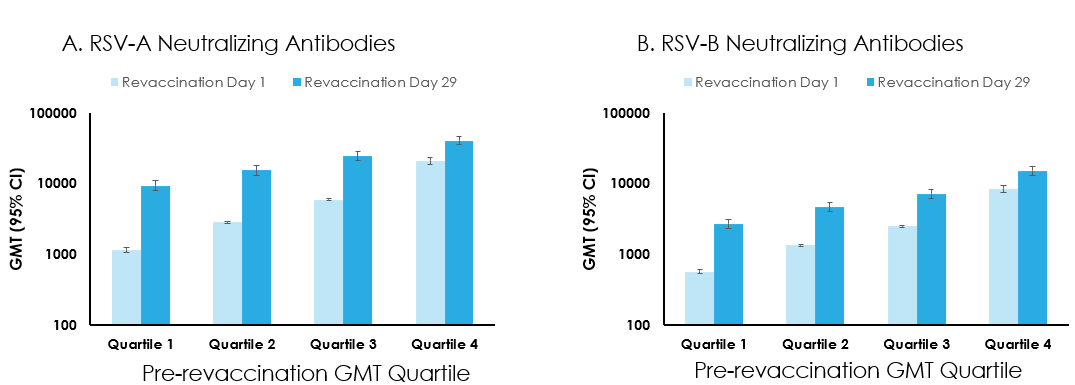


GMT, geometric mean titer; RSV, respiratory syncytial virus

Participants were grouped into quartiles based on their pre-revaccination neutralizing antibody titers. For RSV-A (IU/ml): 1 = 181 to <2028; 2 = ≥2028 to <4113; 3 = ≥4113 to <9582; 4 = ≥9582 to 141709. For RSV-B (IU/ml): 1 = 196 to <965; 2 = ≥965 to <1927; 3 = ≥1927 to <3858; 4 = ≥3858 to 65539

**Supplementary Tables**

**Table S1. Participant Disposition (Enrolled Set)**

|  | **Overall mRNA-1345 revaccination group**  **(N=543)** |
| --- | --- |
| **Received mRNA-1345 revaccination, n (%)** | 543 (100) |
| **Completed the study** | 525 (96.7) |
| **Discontinued from study** | 18 (3.3) |
| **Primary reason for discontinuation, n (%)** |  |
| Adverse event | 3 (0.6) |
| Death | 3 (0.6) |
| Lost to follow-up | 10 (1.8) |
| Non-compliance with study vaccination | 0 |
| Pregnancy | 0 |
| Protocol violation | 0 |
| Solicited adverse reaction/reactogenicity event | 0 |
| Withdrawal of consent by participant | 2 (0.4) |
| Other | 0 |

Percentages are based on the number of participants enrolled in the study.

**Table S2. Number of Days Reporting Solicited Adverse Reactions Within 7 days After mRNA-1345 Primary Vaccination and Overall Revaccination Dose Group (Solicited Safety Set)**

| **Category** | **Overall mRNA-1345 primary vaccination group**  **(N=543)** | **Overall mRNA-1345 revaccination group**  **(N=543)** |
| --- | --- | --- |
| **Solicited adverse reactions, N1** | **541** | **543** |
| Any, n (%) | 338 (62.5) | 338 (62.2) |
| Day of onset |  |  |
| Median (range) | 2.0 (1-7) | 2.0 (1-6) |
| Duration (days) |  |  |
| Median (range) | 3.0 (1-19) | 3.0 (1-37) |
| Persisted beyond 7 days, n (%) | 12 (2.2) | 7 (1.3) |
| **Solicited local adverse reactions, N1** | **541** | **543** |
| Any, n (%) | 297 (54.9) | 303 (55.8) |
| Day of onset |  |  |
| Median (range) | 2.0 (1-7) | 2.0 (1-7) |
| Duration (days) |  |  |
| Median (range) | 2.0 (1-19) | 2.0 (1-9) |
| Persisted beyond 7 days, n (%) | 7 (1.3) | 2 (0.4) |
| **Pain, N1** | **541** | **543** |
| Any, n (%) | 284 (52.5) | 295 (54.3) |
| Day of onset |  |  |
| Median (range) | 2.0 (1-7) | 2.0 (1-5) |
| Duration (days) |  |  |
| Median (range) | 2.0 (1-19) | 2.0 (1-9) |
| Persisted beyond 7 days, n (%) | 5 (0.9) | 1 (0.2) |
| **Erythema, N1** | **541** | **543** |
| Any, n (%) | 16 (3.0) | 13 (2.4) |
| Day of onset |  |  |
| Median (range) | 3.0 (2-7) | 3.0 (1-3) |
| Duration (days) |  |  |
| Median (range) | 2.0 (1-16) | 2.0 (1-7) |
| Persisted beyond 7 days, n (%) | 2 (0.4) | 1 (0.2) |
| **Swelling, N1** | **541** | **543** |
| Any, n (%) | 10 (1.8) | 14 (2.6) |
| Day of onset |  |  |
| Median (range) | 2.0 (1-6) | 2.0 (1-4) |
| Duration (days) |  |  |
| Median (range) | 2.5 (1-19) | 2.0 (1-6) |
| Persisted beyond 7 days, n (%) | 1 (0.2) | 0 |
| **Axillary swelling, N1** | **541** | **543** |
| Any, n (%) | 97 (17.9) | 81 (14.9) |
| Day of onset |  |  |
| Median (range) | 2.0 (1-7) | 2.0 (1-7) |
| Duration (days) |  |  |
| Median (range) | 1.0 (1-7) | 1.0 (1-7) |
| Persisted beyond 7 days, n (%) | 0 | 0 |
| **Solicited systemic adverse reactions, N1** | **541** | **543** |
| Any, n (%) | 250 (46.2) | 273 (50.3) |
| Day of onset |  |  |
| Median | 2.0 (1-7) | 2.0 (1-7) |
| Duration (days) |  |  |
| Median | 2.0 (1-16) | 2.0 (1-36) |
| Persisted beyond 7 days, n (%) | 5 (0.9) | 5 (0.9) |
| **Fever, N1** | **541** | **543** |
| Any, n (%) | 26 (4.8) | 39 (7.2) |
| Day of onset |  |  |
| Median (range) | 2.0 (1-4) | 2.0 (1-5) |
| Duration (days) |  |  |
| Median (range) | 1.0 (1-4) | 1.0 (1-6) |
| Persisted beyond 7 days, n (%) | 0 | 0 |
| **Headache, N1** | **541** | **543** |
| Any, n (%) | 141 (26.1) | 182 (33.5) |
| Day of onset |  |  |
| Median (range) | 2.0 (1-7) | 2.0 (1-7) |
| Duration (days) |  |  |
| Median (range) | 1.0 (1-13) | 1.0 (1-11) |
| Persisted beyond 7 days, n (%) | 3 (0.6) | 2 (0.4) |
| **Fatigue, N1** | **541** | **543** |
| Any, n (%) | 165 (30.5) | 169 (31.1) |
| Day of onset |  |  |
| Median (range) | 2.0 (1-7) | 2.0 (1-7) |
| Duration (days) |  |  |
| Median (range) | 2.0 (1-10) | 2.0 (1-36) |
| Persisted beyond 7 days, n (%) | 3 (0.6) | 3 (0.6) |
| **Myalgia, N1** | **541** | **543** |
| Any, n (%) | 170 (31.4) | 181 (33.3) |
| Day of onset |  |  |
| Median (range) | 2.0 (1-7) | 2.0 (1-7) |
| Duration (days) |  |  |
| Median (range) | 1.0 (1-8) | 1.0 (1-7) |
| Persisted beyond 7 days, n (%) | 1 (0.2) | 0 |
| **Arthralgia, N1** | **541** | **543** |
| Any, n (%) | 132 (24.4) | 156 (28.7) |
| Day of onset |  |  |
| Median (range) | 2.0 (1-7) | 2.0 (1-7) |
| Duration (days) |  |  |
| Median (range) | 2.0 (1-8) | 2.0 (1-16) |
| Persisted beyond 7 days, n (%) | 1 (0.2) | 3 (0.6) |
| **Nausea/vomiting, N1** | **541** | **543** |
| Any, n. (%) | 37 (6.8) | 49 (9.0) |
| Day of onset |  |  |
| Median (range) | 2.0 (1-7) | 2.0 (1-7) |
| Duration (days) |  |  |
| Median (range) | 1.0 (1-4) | 1.0 (1-7) |
| Persisted beyond 7 days, n (%) | 0 | 0 |
| **Chills, N1** | **541** | **543** |
| Any, n (%) | 83 (15.3) | 105 (19.3) |
| Day of onset |  |  |
| Median (range) | 2.0 (1-7) | 2.0 (1-7) |
| Duration (days) |  |  |
| Median (range) | 1.0 (1-7) | 1.0 (1-7) |
| Persisted beyond 7 days, n (%) | 0 | 0 |

Any refers to Grade 1 or above.

N1 is the number of participants receiving revaccination and submitting any data for the event.

Percentages were based on the number of participants receiving injection and submitting any data for the event.

Duration was calculated as the last day − the first day + 1, when the solicited adverse reaction was reported starting within the 7 days of injection.

Symptoms that persisted beyond 7 days were those that were ongoing at Day 7 and persisted, continuously, beyond Day 7.

**Table S3. Summary of Participants with Solicited Adverse Reactions within 7 days After mRNA-1345 Revaccination by Grade and Age Group (Solicited Safety Set)**

| **Solicited adverse reaction category,**  **n (%)** | **Age group:**  **50-59 years** | **Age group:**  **60-74 years** | **Age group:**  ≥**60 years** | **Age group:**  ≥**75 years** | **Overall mRNA-1345 primary vaccination group**  **(N=543)** | **Overall mRNA-1345 revaccination group**  **(N=543)** |
| --- | --- | --- | --- | --- | --- | --- |
| **Solicited adverse reactions, N1** | **210** | **295** | **333** | **38** | **541** | **543** |
| Any | 130 (61.9) | 183 (62.0) | 208 (62.5) | 25 (65.8) | 338 (62.5) | 338 (62.2) |
| Grade 1 | 58 (27.6) | 101 (34.2) | 118 (35.4) | 17 (44.7) | 209 (38.6) | 176 (32.4) |
| Grade 2 | 58 (27.6) | 63 (21.4) | 67 (20.1) | 4 (10.5) | 105 (19.4) | 125 (23.0) |
| Grade 3 | 14 (6.7) | 19 (6.4) | 23 (6.9) | 4 (10.5) | 24 (4.4) | 37 (6.8) |
| Grade 4 | 0 | 0 | 0 | 0 | 0 | 0 |
| **Solicited local adverse reactions, N1** | **210** | **295** | **333** | **38** | **541** | **543** |
| Any | 120 (57.1) | 160 (54.2) | 183 (55.0) | 23 (60.5) | 297 (54.9) | 303 (55.8) |
| Grade 1 | 76 (36.2) | 112 (38.0) | 130 (39.0) | 18 (47.4) | 240 (44.4) | 206 (37.9) |
| Grade 2 | 39 (18.6) | 40 (13.6) | 43 (12.9) | 3 (7.9) | 50 (9.2) | 82 (15.1) |
| Grade 3 | 5 (2.4) | 8 (2.7) | 10 (3.0) | 2 (5.3) | 7 (1.3) | 15 (2.8) |
| Grade 4 | 0 | 0 | 0 | 0 | 0 | 0 |
| Pain, N1 | 210 | 295 | 333 | 38 | 541 | 543 |
| Any | 117 (55.7) | 155 (52.5) | 178 (53.5) | 23 (60.5) | 284 (52.5) | 295 (54.3) |
| Grade 1 | 77 (36.7) | 116 (39.3) | 134 (40.2) | 18 (47.4) | 236 (43.6) | 211 (38.9) |
| Grade 2 | 38 (18.1) | 38 (12.9) | 42 (12.6) | 4 (10.5) | 43 (7.9) | 80 (14.7) |
| Grade 3 | 2 (1.0) | 1 (0.3) | 2 (0.6) | 1 (2.6) | 5 (0.9) | 4 (0.7) |
| Grade 4 | 0 | 0 | 0 | 0 | 0 | 0 |
| Erythema (redness), N1 | 210 | 295 | 333 | 38 | 541 | 543 |
| Any | 5 (2.4) | 8 (2.7) | 8 (2.4) | 0 | 16 (3.0) | 13 (2.4) |
| Grade 1 | 1 (0.5) | 3 (1.0) | 3 (0.9) | 0 | 6 (1.1) | 4 (0.7) |
| Grade 2 | 2 (1.0) | 1 (0.3) | 1 (0.3) | 0 | 8 (1.5) | 3 (0.6) |
| Grade 3 | 2 (1.0) | 4 (1.4) | 4 (1.2) | 0 | 2 (0.4) | 6 (1.1) |
| Grade 4 | 0 | 0 | 0 | 0 | 0 | 0 |
| Swelling (hardness) – N1 | 210 | 295 | 333 | 38 | 541 | 543 |
| Any | 7 (3.3) | 7 (2.4) | 7 (2.1) | 0 | 10 (1.8) | 14 (2.6) |
| Grade 1 | 4 (1.9) | 3 (1.0) | 3 (0.9) | 0 | 5 (0.9) | 7 (1.3) |
| Grade 2 | 2 (1.0) | 1 (0.3) | 1 (0.3) | 0 | 5 (0.9) | 3 (0.6) |
| Grade 3 | 1 (0.5) | 3 (1.0) | 3 (0.9) | 0 | 0 | 4 (0.7) |
| Grade 4 | 0 | 0 | 0 | 0 | 0 | 0 |
| Axillary (underarm) swelling or tenderness, N1 | 210 | 295 | 333 | 38 | 541 | 543 |
| Any | 31 (14.8) | 40 (13.6) | 50 (15.0) | 10 (26.3) | 97 (17.9) | 81 (14.9) |
| Grade 1 | 21 (10.0) | 31 (10.5) | 40 (12.0) | 9 (23.7) | 80 (14.8) | 61 (11.2) |
| Grade 2 | 9 (4.3) | 7 (2.4) | 7 (2.1) | 0 | 16 (3.0) | 16 (2.9) |
| Grade 3 | 1 (0.5) | 2 (0.7) | 3 (0.9) | 1 (2.6) | 1 (0.2) | 4 (0.7) |
| Grade 4 | 0 | 0 | 0 | 0 | 0 | 0 |
| **Solicited systemic adverse reactions, N1** | **210** | **295** | **333** | **38** | **541** | **543** |
| Any | 110 (52.4) | 144 (48.8) | 163 (48.9) | 19 (50.0) | 130 (51.2) | 273 (50.3) |
| Grade 1 | 46 (21.9) | 75 (25.4) | 88 (26.4) | 13 (34.2) | 62 (24.4) | 134 (24.7) |
| Grade 2 | 52 (24.8) | 57 (19.3) | 61 (18.3) | 4 (10.5) | 54 (21.3) | 113 (20.8) |
| Grade 3 | 12 (5.7) | 12 (4.1) | 14 (4.2) | 2 (5.3) | 14 (5.5) | 26 (4.8) |
| Grade 4 | 0 | 0 | 0 | 0 | 0 | 0 |
| Fever, N1 | 210 | 295 | 333 | 38 | 541 | 543 |
| Any | 13 (6.2) | 24 (8.1) | 26 (7.8) | 2 (5.3) | 26 (4.8) | 39 (7.2) |
| Grade 1 | 8 (3.8) | 17 (5.8) | 18 (5.4) | 1 (2.6) | 16 (3.0) | 26 (4.8) |
| Grade 2 | 3 (1.4) | 6 (2.0) | 7 (2.1) | 1 (2.6) | 8 (1.5) | 10 (1.8) |
| Grade 3 | 2 (1.0) | 1 (0.3) | 1 (0.3) | 0 | 2 (0.4) | 3 (0.6) |
| Grade 4 | 0 | 0 | 0 | 0 | 0 | 0 |
| Headache – N1 | 210 | 295 | 333 | 38 | 541 | 543 |
| Any | 82 (39.0) | 88 (29.8) | 100 (30.0) | 12 (31.6) | 141 (26.1) | 182 (33.5) |
| Grade 1 | 49 (23.3) | 52 (17.6) | 60 (18.0) | 8 (21.1) | 108 (20.0) | 109 (20.1) |
| Grade 2 | 28 (13.3) | 33 (11.2) | 37 (11.1) | 4 (10.5) | 30 (5.5) | 65 (12.0) |
| Grade 3 | 5 (2.4) | 3 (1.0) | 3 (0.9) | 0 | 3 (0.6) | 8 (1.5) |
| Grade 4 | 0 | 0 | 0 | 0 | 0 | 0 |
| Fatigue, N1 | 210 | 295 | 333 | 38 | 541 | 543 |
| Any | 69 (32.9) | 86 (29.2) | 100 (30.0) | 14 (36.8) | 165 (30.5) | 169 (31.1) |
| Grade 1 | 27 (12.9) | 44 (14.9) | 52 (15.6) | 8 (21.1) | 99 (18.3) | 79 (14.5) |
| Grade 2 | 34 (16.2) | 35 (11.9) | 40 (12.0) | 5 (13.2) | 56 (10.4) | 74 (13.6) |
| Grade 3 | 8 (3.8) | 7 (2.4) | 8 (2.4) | 1 (2.6) | 10 (1.8) | 16 (2.9) |
| Grade 4 | 0 | 0 | 0 | 0 | 0 | 0 |
| Myalgia, N1 | 210 | 295 | 333 | 38 | 541 | 543 |
| Any | 79 (37.6) | 90 (30.5) | 102 (30.6) | 12 (31.6) | 170 (31.4) | 181 (33.3) |
| Grade 1 | 33 (15.7) | 49 (16.6) | 57 (17.1) | 8 (21.1) | 98 (18.1) | 90 (16.6) |
| Grade 2 | 41 (19.5) | 34 (11.5) | 38 (11.4) | 4 (10.5) | 59 (10.9) | 79 (14.5) |
| Grade 3 | 5 (2.4) | 7 (2.4) | 7 (2.1) | 0 | 13 (2.4) | 12 (2.2) |
| Grade 4 | 0 | 0 | 0 | 0 | 0 | 0 |
| Arthralgia, N1 | 210 | 295 | 333 | 38 | 541 | 543 |
| Any | 65 (31.0) | 81 (27.5) | 91 (27.3) | 10 (26.3) | 132 (24.4) | 156 (28.7) |
| Grade 1 | 33 (15.7) | 51 (17.3) | 58 (17.4) | 7 (18.4) | 78 (14.4) | 91 (16.8) |
| Grade 2 | 28 (13.3) | 22 (7.5) | 25 (7.5) | 3 (7.9) | 47 (8.7) | 53 (9.8) |
| Grade 3 | 4 (1.9) | 8 (2.7) | 8 (2.4) | 0 | 7 (1.3) | 12 (2.2) |
| Grade 4 | 0 | 0 | 0 | 0 | 0 | 0 |
| Nausea/vomiting, N1 | 210 | 295 | 333 | 38 | 541 | 543 |
| Any | 20 (9.5) | 26 (8.8) | 29 (8.7) | 3 (7.9) | 37 (6.8) | 49 (9.0) |
| Grade 1 | 15 (7.1) | 20 (6.8) | 21 (6.3) | 1 (2.6) | 31 (5.7) | 36 (6.6) |
| Grade 2 | 5 (2.4) | 6 (2.0) | 8 (2.4) | 2 (5.3) | 6 (1.1) | 13 (2.4) |
| Grade 3 | 0 | 0 | 0 | 0 | 0 | 0 |
| Grade 4 | 0 | 0 | 0 | 0 | 0 | 0 |
| Chills, N1 | 210 | 295 | 333 | 38 | 541 | 543 |
| Any | 47 (22.4) | 54 (18.3) | 58 (17.4) | 4 (10.5) | 83 (15.3) | 105 (19.3) |
| Grade 1 | 18 (8.6) | 27 (9.2) | 28 (8.4) | 1 (2.6) | 45 (8.3) | 46 (8.5) |
| Grade 2 | 27 (12.9) | 26 (8.8) | 28 (8.4) | 2 (5.3) | 35 (6.5) | 55 (10.1) |
| Grade 3 | 2 (1.0) | 1 (0.3) | 2 (0.6) | 1 (2.6) | 3 (0.6) | 4 (0.7) |
| Grade 4 | 0 | 0 | 0 | 0 | 0 | 0 |

Any is Grade 1 or above.

N1 is the number of participants receiving revaccination and submitting any data for the event.

Percentages are based on the number of participants receiving revaccination injection and submitting any data for the event.

**Table S4. Overall Summary of Unsolicited Adverse Events up to Data Cutoff (Safety Set)**

|  | |  |
| --- | --- | --- |
|  | | **Overall mRNA-1345 revaccination group**  **(N=543)** |
| **Unsolicited AEs up to 28 days after revaccination, regardless of relationship to study vaccination, n (%)** |  |  |
| Any | | 31 (5.7) |
| Serious | | 3 (0.6) |
| Fatal | | 0 |
| Medically attended | | 21 (3.9) |
| Leading to study discontinuation | | 0 |
| Severe | | 2 (0.4) |
| Non-serious | | 28 (5.2) |
| Of special interest | | 1 (0.2) |
| **Unsolicited AEs up to 28 days after revaccination, related to study vaccination, n (%)** |  |  |
| Any | | 5 (0.9) |
| Serious | | 0 |
| Fatal | | 0 |
| Medically attended | | 2 (0.4) |
| Leading to study discontinuation | | 0 |
| Severe | | 0 |
| Non-serious | | 5 (0.9) |
| Of special interest | | 0 |
| **Unsolicited AEs up to data cutoff, regardless of relationship to study vaccination, n (%)** |  |  |
| Serious | | 28 (5.2) |
| Fatal | | 3 (0.6) |
| Medically attended | | 105 (19.3) |
| Leading to study discontinuation | | 5 (0.9) |
| Of special interest | | 2 (0.4) |
| **Unsolicited AEs up to data cutoff, related to study vaccination, n (%)** |  |  |
| Serious | | 0 |
| Fatal | | 0 |
| Medically attended | | 2 (0.4) |
| Leading to study discontinuation | | 0 |
| AEs of special interest | | 0 |

Abbreviation: AE, adverse event.

Percentages are based on the number of participants in the safety set.

**Table S5. Summary of RSV-A and RSV-B Neutralizing Antibody Titers (IU/mL) and Seroresponse Rates of mRNA-1345 Revaccination Versus Primary Dose (Per-Protocol Set)**

|  | **Overall mRNA-1345 revaccination group**  (N=525) |
| --- | --- |
| **RSV-A** |  |
| **Primary dose Day 29, N1** | 519 |
| GMT (95% CI)^a^ | 18190.30 (16467.13, 20093.79) |
| Seroresponse,^b^ n (%) | 390 (75.3) |
| 95% CI | 71.3, 78.9 |
| **Revaccination Day 29, N1** | 525 |
| GMT (95% CI)^a^ | 19649.18 (18017.92, 21428.12) |
| Seroresponse,^b^ n (%) | 406 (77.5) |
| 95% CI | 73.7, 81.0 |
| **Revaccination Day 29 vs. primary dose Day 29, N2** | 518 |
| GMR^c^ (95% CI) | 1.08 (1.00, 1.17) |
| Seroresponse rate difference,^d^ % (95% CI) | 2.1 (-1.7, 5.9) |
| **RSV-B** |  |
| **Primary dose Day 29, N1** | 524 |
| GMT (95% CI)^a^ | 6746.41 (6136.32, 7417.16) |
| Seroresponse,^b^ n (%) | 252 (48.2) |
| 95% CI | 43.8, 52.6 |
| **Revaccination Day 29, N1** | 525 |
| GMT (95% CI)^a^ | 6123.47 (5599.77, 6696.14) |
| Seroresponse,^b^ n (%) | 249 (47.5) |
| 95% CI | 43.2, 51.9 |
| **Revaccination Day 29 vs. primary dose Day 29, N2** | 523 |
| GMR^c^ (95% CI) | 0.91 (0.84, 0.98) |
| Seroresponse rate difference,^d^ % (95% CI) | -0.6 (-5.0, 3.9) |

Abbreviations: CI, confidence interval; GM, geometric mean; GMT, geometric mean titer; GMR, geometric mean ratio; LLOQ, lower limit of quantification; nAb, neutralizing antibody titers; RSV, respiratory syncytial virus; ULOQ, upper limit of quantification.

Antibody values reported as below the LLOQ are replaced by 0.5 x LLOQ. Values greater than the ULOQ are replaced by the ULOQ.

N1 is the number of participants with non-missing antibody data at the corresponding timepoint.

N2 is the number of participants with non-missing antibody data at baseline (primary dose Day 1), Day 29 and revaccination Day 29.

^a^95% CI was calculated based on the t-distribution of the log-transformed values then back transformed to the original scale for presentation.

^b^Seroresponse at a participant level was defined as a change from below the LLOQ to equal or above 4x LLOQ, or at least a 4-fold increase if baseline is equal to or above the LLOQ. 95% CI is calculated using the Clopper-Pearson method.

^c^GMR and 95% CI were calculated by back transforming the mean and 95% CI of paired difference of nAbs on the logarithmic scale (based on t-distribution) between revaccination Day 29 and Day 29 following primary dose.

^d^The difference of seroresponse rate and 95% CI were estimated through a linear probability model with repeated measures.

**Table S6. Summary of RSV-A and RSV-B Neutralizing Antibody Titers (IU/mL) and Seroresponse Rates (Per-Protocol Set)**

|  | **Overall mRNA-1345 revaccination group**  **(N=525)** |
| --- | --- |
| **RSV-A** |  |
| **Baseline (Day 1), n** | 524 |
| GMT (95% CI)^a^ | 2155.15 (1945.28, 2387.66) |
| **Primary dose Day 29, n** | 519 |
| GMT (95% CI)^a^ | 18190.30 (16467.13, 20093.79) |
| GM fold-rise (95% CI)^a^ | 8.46 (7.63, 9.38) |
| **Revaccination Day 1, n** | 525 |
| GMT (95% CI)^a^ | 4537.34 (4110.83, 5008.10) |
| GM fold-rise (95% CI)^a^ | 2.10 (1.92, 2.30) |
| **Revaccination Day 29, n** | 525 |
| GMT (95% CI)^a^ | 19649.18 (18017.92, 21428.12) |
| GM fold-rise (95% CI)^a^ | 9.11 (8.26, 10.04) |
| Seroresponse rate^b^, n (%)^c^ (95% CI)^d^ | 406 (77.5) (73.7, 81.0) |
| ≥2-fold increase from baseline^e^, n (%) ^c^ (95% CI)^d^ | 480 (91.6) (88.9, 93.8) |
| **RSV-B** |  |
| **Baseline (Day 1), n** | 524 |
| GMT (95% CI)^a^ | 1624.18 (1468.92, 1795.85) |
| **Primary Dose Day 29, n** | 524 |
| GMT (95% CI)^a^ | 6746.41 (6136.32, 7417.16) |
| GM fold-rise (95% CI)^a^ | 4.16 (3.78, 4.57) |
| **Revaccination Day 1, n** | 525 |
| GMT (95% CI)^a^ | 2016.14 (1838.96, 2210.39) |
| GM fold-rise (95% CI)^a^ | 1.24 (1.15, 1.34) |
| **Revaccination Day 29, n** | 525 |
| GMT (95% CI)^a^ | 6123.47 (5599.77, 6696.14) |
| GM fold-rise (95% CI)^a^ | 3.77 (3.43, 4.14) |
| Seroresponse rate^b^, n (%)^c^ (95% CI)^d^ | 249 (47.5) (43.2, 51.9) |
| ≥2-fold increase from baseline^e^, n (%)^c^ (95% CI)^d^ | 366 (69.8) (65.7, 73.8) |

Abbreviations: CI, confidence interval; GM, geometric mean; GMT, geometric mean titer; LLOQ, lower limit of quantitation; ULOQ, upper limit of quantitation.

N1 is the number of participants with non-missing data at baseline (primary dose Day 1) and the corresponding after baseline timepoint.

Antibody values reported as below the LLOQ were replaced by 0.5 x LLOQ. Values greater than the ULOQ were replaced by the ULOQ.

^a^95% CI was calculated based on the *t*-distribution of the log-transformed values or the difference in the log-transformed values for GMT and GM fold-rise, respectively, then back transformed to the original scale for presentation.

^b^Seroresponse at a participant level was defined as a change from below the LLOQ to equal or above 4 x LLOQ, or at least a 4-fold increase if baseline is equal to or above the LLOQ.

^c^Number of participants meeting the criterion at the time point. Percentages were based on N1.

^d^95% CI was calculated using the Clopper-Pearson method.

^e^≥2-fold increase from baseline at participant level was defined as a change from below the LLOQ to equal or above 2 x LLOQ, or at least a 2-fold increase if baseline was equal to or above the LLOQ.
